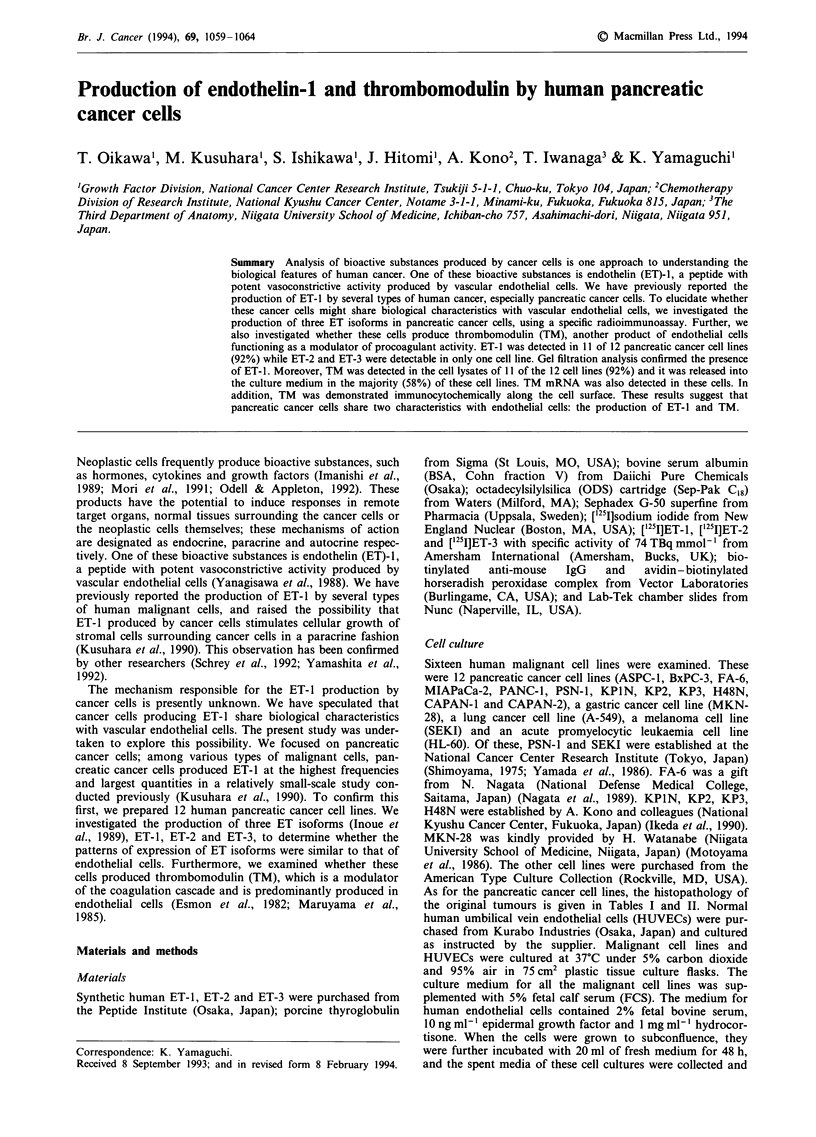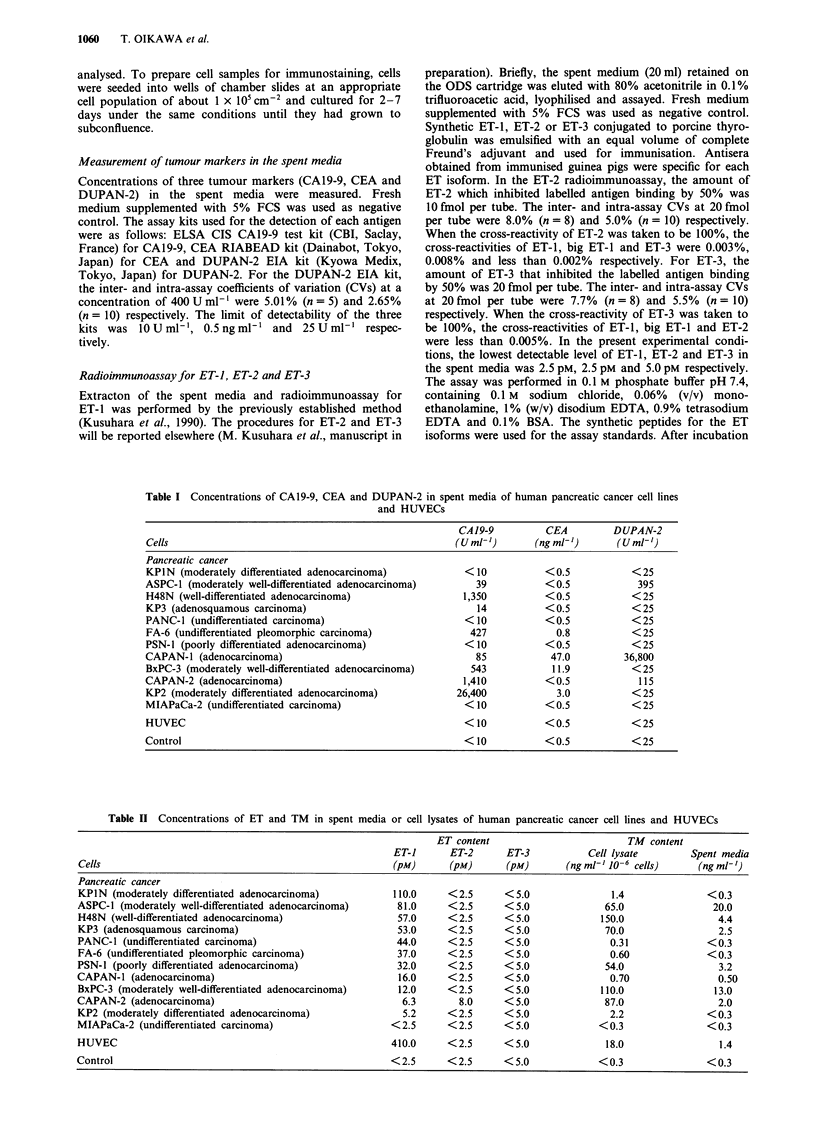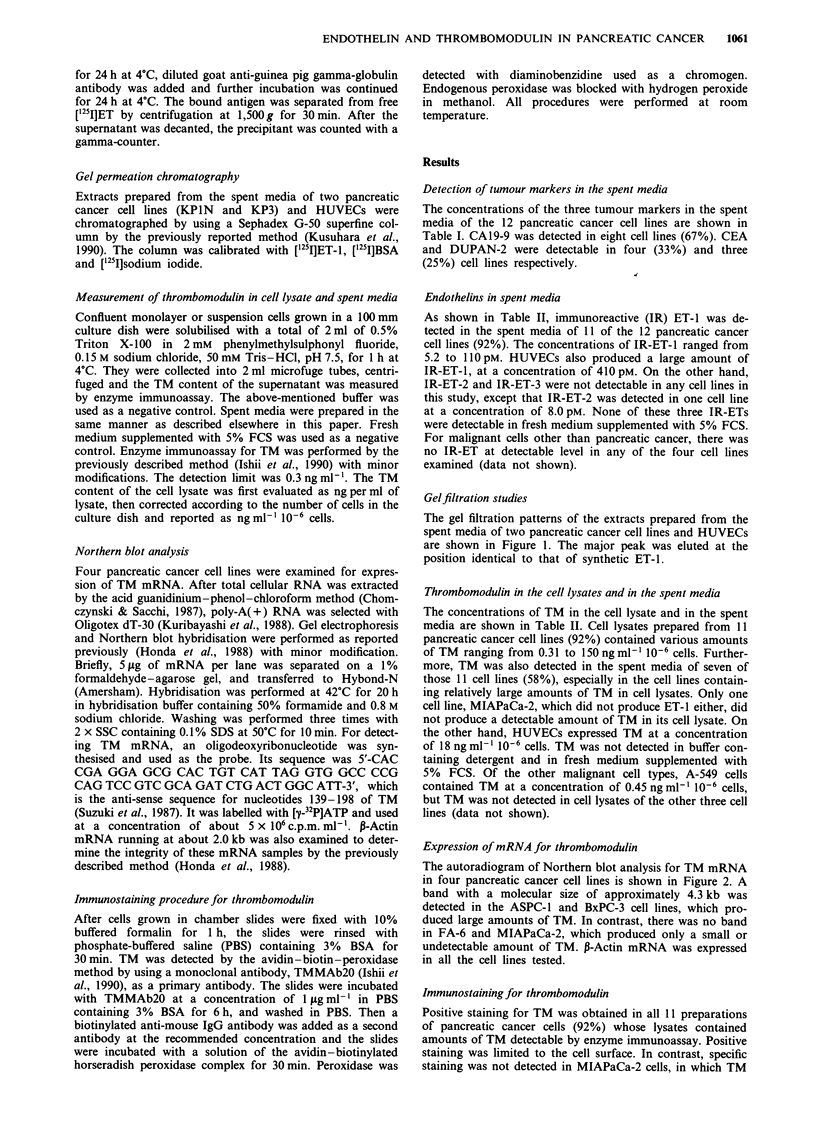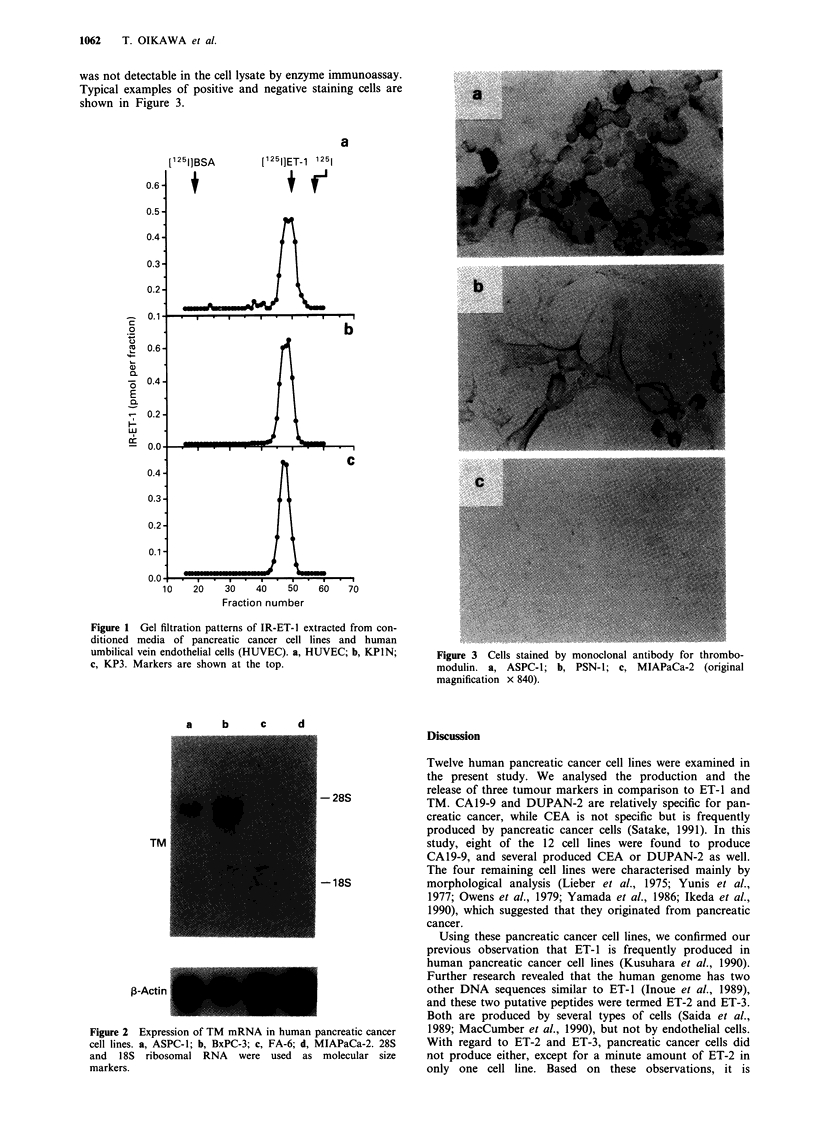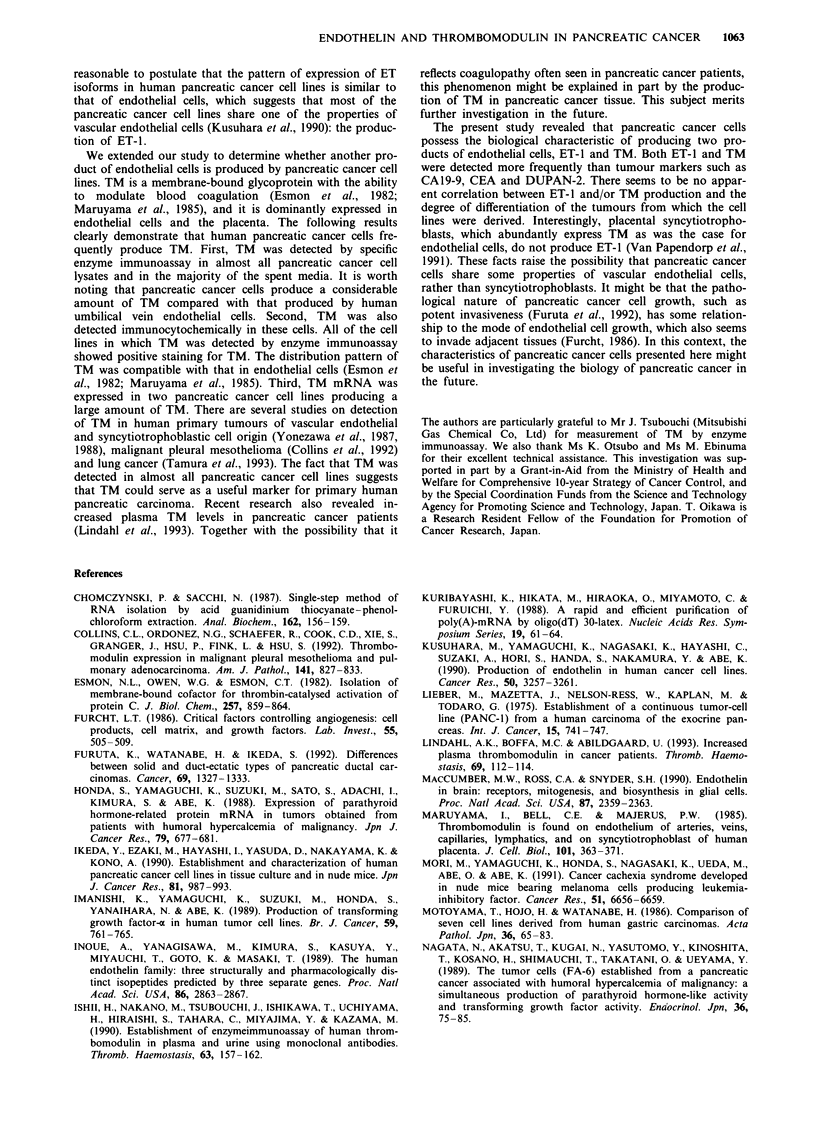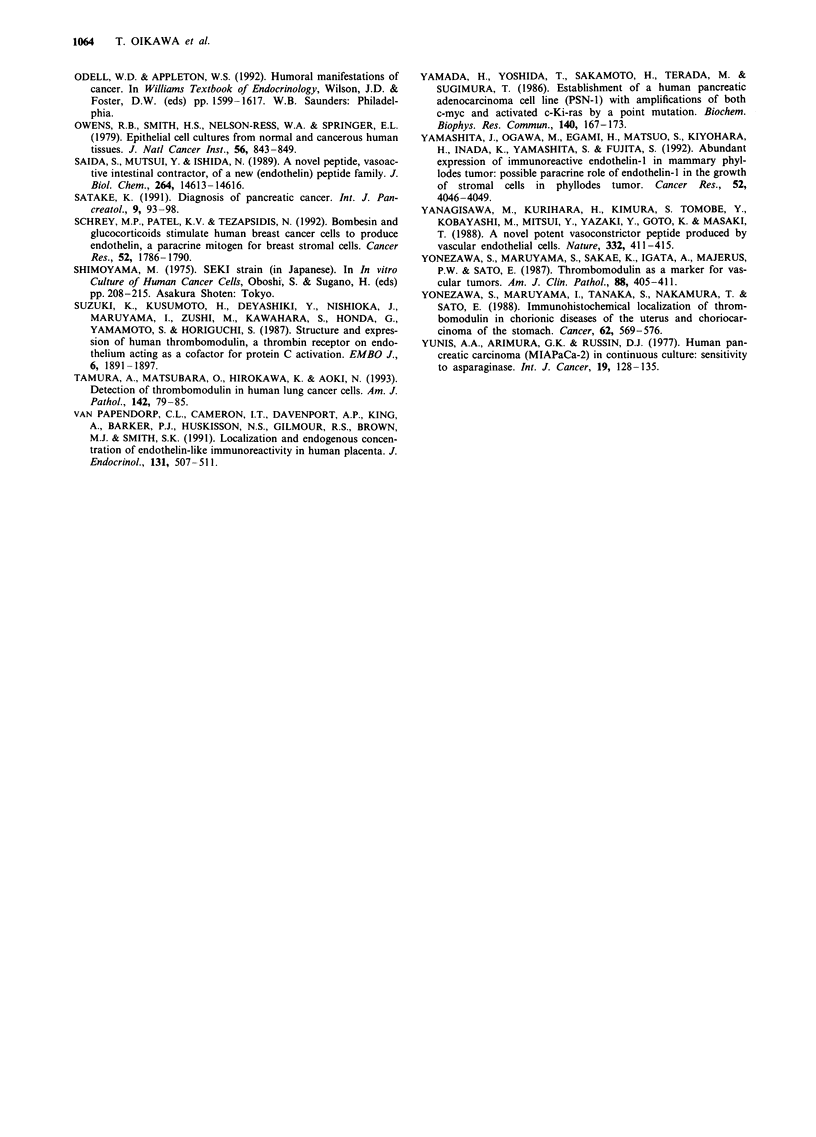# Production of endothelin-1 and thrombomodulin by human pancreatic cancer cells.

**DOI:** 10.1038/bjc.1994.208

**Published:** 1994-06

**Authors:** T. Oikawa, M. Kushuhara, S. Ishikawa, J. Hitomi, A. Kono, T. Iwanaga, K. Yamaguchi

**Affiliations:** Growth Factor Division, National Cancer Center Research Institute, Tokyo, Japan.

## Abstract

**Images:**


Br. J. Cancer (1994), 69, 1059–1064 © Macmillan Press Ltd., 1994

### Production of endothelin-1 and thrombomodulin by human pancreatic cancer cells

T. Oikawa[1], M. Kusuhara[1], S. Ishikawa[1], J. Hitomi[1], A. Kono[2], T. Iwanaga[3] & K. Yamaguchi[1]

[1]*Growth Factor Division, National Cancer Center Research Institute, Tsukiji 5-1-1, Chuo-ku, Tokyo 104, Japan;* [2]*Chemotherapy Division of Research Institute, National Kyushu Cancer Center, Notame 3-1-1, Minami-ku, Fukuoka, Fukuoka 815, Japan;* [3]*The Third Department of Anatomy, Niigata University School of Medicine, Ichiban-cho 757, Asahimachi-dori, Niigata, Niigata 951, Japan.*

**Summary** Analysis of bioactive substances produced by cancer cells is one approach to understanding the biological features of human cancer. One of these bioactive substances is endothelin (ET)-1, a peptide with potent vasoconstrictive activity produced by vascular endothelial cells. We have previously reported the production of ET-1 by several types of human cancer, especially pancreatic cancer cells. To elucidate whether these cancer cells might share biological characteristics with vascular endothelial cells, we investigated the production of three ET isoforms in pancreatic cancer cells, using a specific radioimmunoassay. Further, we also investigated whether these cells produce thrombomodulin (TM), another product of endothelial cells functioning as a modulator of procoagulant activity. ET-1 was detected in 11 of 12 pancreatic cancer cell lines (92%) while ET-2 and ET-3 were detectable in only one cell line. Gel filtration analysis confirmed the presence of ET-1. Moreover, TM was detected in the cell lysates of 11 of the 12 cell lines (92%) and it was released into the culture medium in the majority (58%) of these cell lines. TM mRNA was also detected in these cells. In addition, TM was demonstrated immunocytochemically along the cell surface. These results suggest that pancreatic cancer cells share two characteristics with endothelial cells: the production of ET-1 and TM.

Neoplastic cells frequently produce bioactive substances, such as hormones, cytokines and growth factors (Imanishi *et al.*, 1989; Mori *et al.*, 1991; Odell & Appleton, 1992). These products have the potential to induce responses in remote target organs, normal tissues surrounding the cancer cells or the neoplastic cells themselves; these mechanisms of action are designated as endocrine, paracrine and autocrine respectively. One of these bioactive substances is endothelin (ET)-1, a peptide with potent vasoconstrictive activity produced by vascular endothelial cells (Yanagisawa *et al.*, 1988). We have previously reported the production of ET-1 by several types of human malignant cells, and raised the possibility that ET-1 produced by cancer cells stimulates cellular growth of stromal cells surrounding cancer cells in a paracrine fashion (Kusuhara *et al.*, 1990). This observation has been confirmed by other researchers (Schrey *et al.*, 1992; Yamashita *et al.*, 1992).

The mechanism responsible for the ET-1 production by cancer cells is presently unknown. We have speculated that cancer cells producing ET-1 share biological characteristics with vascular endothelial cells. The present study was undertaken to explore this possibility. We focused on pancreatic cancer cells; among various types of malignant cells, pancreatic cancer cells produced ET-1 at the highest frequencies and largest quantities in a relatively small-scale study conducted previously (Kusuhara *et al.*, 1990). To confirm this first, we prepared 12 human pancreatic cancer cell lines. We investigated the production of three ET isoforms (Inoue *et al.*, 1989), ET-1, ET-2 and ET-3, to determine whether the patterns of expression of ET isoforms were similar to that of endothelial cells. Furthermore, we examined whether these cells produced thrombomodulin (TM), which is a modulator of the coagulation cascade and is predominantly produced in endothelial cells (Esmon *et al.*, 1982; Maruyama *et al.*, 1985).

### Materials and methods

### *Materials*

Synthetic human ET-1, ET-2 and ET-3 were purchased from the Peptide Institute (Osaka, Japan); porcine thyroglobulin from Sigma (St Louis, MO, USA); bovine serum albumin (BSA, Cohn fraction V) from Daiichi Pure Chemicals (Osaka); octadecylsilylsilica (ODS) cartridge (Sep-Pak $C_{18}$) from Waters (Milford, MA); Sephadex G-50 superfine from Pharmacia (Uppsala, Sweden); [$^{125}$I]sodium iodide from New England Nuclear (Boston, MA, USA); [$^{125}$I]ET-1, [$^{125}$I]ET-2 and [$^{125}$I]ET-3 with specific activity of 74 TBq mmol$^{-1}$ from Amersham International (Amersham, Bucks, UK); biotinylated anti-mouse IgG and avidin–biotinylated horseradish peroxidase complex from Vector Laboratories (Burlingame, CA, USA); and Lab-Tek chamber slides from Nunc (Naperville, IL, USA).

### *Cell culture*

Sixteen human malignant cell lines were examined. These were 12 pancreatic cancer cell lines (ASPC-1, BxPC-3, FA-6, MIAPaCa-2, PANC-1, PSN-1, KP1N, KP2, KP3, H48N, CAPAN-1 and CAPAN-2), a gastric cancer cell line (MKN-28), a lung cancer cell line (A-549), a melanoma cell line (SEKI) and an acute promyelocytic leukaemia cell line (HL-60). Of these, PSN-1 and SEKI were established at the National Cancer Center Research Institute (Tokyo, Japan) (Shimoyama, 1975; Yamada *et al.*, 1986). FA-6 was a gift from N. Nagata (National Defense Medical College, Saitama, Japan) (Nagata *et al.*, 1989). KP1N, KP2, KP3, H48N were established by A. Kono and colleagues (National Kyushu Cancer Center, Fukuoka, Japan) (Ikeda *et al.*, 1990). MKN-28 was kindly provided by H. Watanabe (Niigata University School of Medicine, Niigata, Japan) (Motoyama *et al.*, 1986). The other cell lines were purchased from the American Type Culture Collection (Rockville, MD, USA). As for the pancreatic cancer cell lines, the histopathology of the original tumours is given in Tables I and II. Normal human umbilical vein endothelial cells (HUVECs) were purchased from Kurabo Industries (Osaka, Japan) and cultured as instructed by the supplier. Malignant cell lines and HUVECs were cultured at 37°C under 5% carbon dioxide and 95% air in 75 cm$^2$ plastic tissue culture flasks. The culture medium for all the malignant cell lines was supplemented with 5% fetal calf serum (FCS). The medium for human endothelial cells contained 2% fetal bovine serum, 10 ng ml$^{-1}$ epidermal growth factor and 1 mg ml$^{-1}$ hydrocortisone. When the cells were grown to subconfluence, they were further incubated with 20 ml of fresh medium for 48 h, and the spent media of these cell cultures were collected and

Correspondence: K. Yamaguchi.
Received 8 September 1993; and in revised form 8 February 1994.

analysed. To prepare cell samples for immunostaining, cells were seeded into wells of chamber slides at an appropriate cell population of about $1 \times 10^5 cm^{-2}$ and cultured for 2–7 days under the same conditions until they had grown to subconfluence.

*Measurement of tumour markers in the spent media*

Concentrations of three tumour markers (CA19-9, CEA and DUPAN-2) in the spent media were measured. Fresh medium supplemented with 5% FCS was used as negative control. The assay kits used for the detection of each antigen were as follows: ELSA CIS CA19-9 test kit (CBI, Saclay, France) for CA19-9, CEA RIABEAD kit (Dainabot, Tokyo, Japan) for CEA and DUPAN-2 EIA kit (Kyowa Medix, Tokyo, Japan) for DUPAN-2. For the DUPAN-2 EIA kit, the inter- and intra-assay coefficients of variation (CVs) at a concentration of $400 U ml^{-1}$ were 5.01% ($n = 5$) and 2.65% ($n = 10$) respectively. The limit of detectability of the three kits was $10 U ml^{-1}$, $0.5 ng ml^{-1}$ and $25 U ml^{-1}$ respectively.

*Radioimmunoassay for ET-1, ET-2 and ET-3*

Extracton of the spent media and radioimmunoassay for ET-1 was performed by the previously established method (Kusuhara *et al.*, 1990). The procedures for ET-2 and ET-3 will be reported elsewhere (M. Kusuhara *et al.*, manuscript in preparation). Briefly, the spent medium (20 ml) retained on the ODS cartridge was eluted with 80% acetonitrile in 0.1% trifluoroacetic acid, lyophilised and assayed. Fresh medium supplemented with 5% FCS was used as negative control. Synthetic ET-1, ET-2 or ET-3 conjugated to porcine thyroglobulin was emulsified with an equal volume of complete Freund's adjuvant and used for immunisation. Antisera obtained from immunised guinea pigs were specific for each ET isoform. In the ET-2 radioimmunoassay, the amount of ET-2 which inhibited labelled antigen binding by 50% was 10 fmol per tube. The inter- and intra-assay CVs at 20 fmol per tube were 8.0% ($n = 8$) and 5.0% ($n = 10$) respectively. When the cross-reactivity of ET-2 was taken to be 100%, the cross-reactivities of ET-1, big ET-1 and ET-3 were 0.003%, 0.008% and less than 0.002% respectively. For ET-3, the amount of ET-3 that inhibited the labelled antigen binding by 50% was 20 fmol per tube. The inter- and intra-assay CVs at 20 fmol per tube were 7.7% ($n = 8$) and 5.5% ($n = 10$) respectively. When the cross-reactivity of ET-3 was taken to be 100%, the cross-reactivities of ET-1, big ET-1 and ET-2 were less than 0.005%. In the present experimental conditions, the lowest detectable level of ET-1, ET-2 and ET-3 in the spent media was 2.5 pM, 2.5 pM and 5.0 pM respectively. The assay was performed in 0.1 M phosphate buffer pH 7.4, containing 0.1 M sodium chloride, 0.06% (v/v) monoethanolamine, 1% (w/v) disodium EDTA, 0.9% tetrasodium EDTA and 0.1% BSA. The synthetic peptides for the ET isoforms were used for the assay standards. After incubation

**Table I** Concentrations of CA19-9, CEA and DUPAN-2 in spent media of human pancreatic cancer cell lines and HUVECs

| *Cells* | *CA19-9 ($U ml^{-1}$)* | *CEA ($ng ml^{-1}$)* | *DUPAN-2 ($U ml^{-1}$)* |
|---|---|---|---|
| *Pancreatic cancer* | | | |
| KP1N (moderately differentiated adenocarcinoma) | <10 | <0.5 | <25 |
| ASPC-1 (moderately well-differentiated adenocarcinoma) | 39 | <0.5 | 395 |
| H48N (well-differentiated adenocarcinoma) | 1,350 | <0.5 | <25 |
| KP3 (adenosquamous carcinoma) | 14 | <0.5 | <25 |
| PANC-1 (undifferentiated carcinoma) | <10 | <0.5 | <25 |
| FA-6 (undifferentiated pleomorphic carcinoma) | 427 | 0.8 | <25 |
| PSN-1 (poorly differentiated adenocarcinoma) | <10 | <0.5 | <25 |
| CAPAN-1 (adenocarcinoma) | 85 | 47.0 | 36,800 |
| BxPC-3 (moderately well-differentiated adenocarcinoma) | 543 | 11.9 | <25 |
| CAPAN-2 (adenocarcinoma) | 1,410 | <0.5 | 115 |
| KP2 (moderately differentiated adenocarcinoma) | 26,400 | 3.0 | <25 |
| MIAPaCa-2 (undifferentiated carcinoma) | <10 | <0.5 | <25 |
| HUVEC | <10 | <0.5 | <25 |
| Control | <10 | <0.5 | <25 |

**Table II** Concentrations of ET and TM in spent media or cell lysates of human pancreatic cancer cell lines and HUVECs

| *Cells* | *ET content* *ET-1 (pM)* | *ET-2 (pM)* | *ET-3 (pM)* | *TM content* *Cell lysate ($ng ml^{-1} 10^{-6}$ cells)* | *Spent media ($ng ml^{-1}$)* |
|---|---|---|---|---|---|
| *Pancreatic cancer* | | | | | |
| KP1N (moderately differentiated adenocarcinoma) | 110.0 | <2.5 | <5.0 | 1.4 | <0.3 |
| ASPC-1 (moderately well-differentiated adenocarcinoma) | 81.0 | <2.5 | <5.0 | 65.0 | 20.0 |
| H48N (well-differentiated adenocarcinoma) | 57.0 | <2.5 | <5.0 | 150.0 | 4.4 |
| KP3 (adenosquamous carcinoma) | 53.0 | <2.5 | <5.0 | 70.0 | 2.5 |
| PANC-1 (undifferentiated carcinoma) | 44.0 | <2.5 | <5.0 | 0.31 | <0.3 |
| FA-6 (undifferentiated pleomorphic carcinoma) | 37.0 | <2.5 | <5.0 | 0.60 | <0.3 |
| PSN-1 (poorly differentiated adenocarcinoma) | 32.0 | <2.5 | <5.0 | 54.0 | 3.2 |
| CAPAN-1 (adenocarcinoma) | 16.0 | <2.5 | <5.0 | 0.70 | 0.50 |
| BxPC-3 (moderately well-differentiated adenocarcinoma) | 12.0 | <2.5 | <5.0 | 110.0 | 13.0 |
| CAPAN-2 (adenocarcinoma) | 6.3 | 8.0 | <5.0 | 87.0 | 2.0 |
| KP2 (moderately differentiated adenocarcinoma) | 5.2 | <2.5 | <5.0 | 2.2 | <0.3 |
| MIAPaCa-2 (undifferentiated carcinoma) | <2.5 | <2.5 | <5.0 | <0.3 | <0.3 |
| HUVEC | 410.0 | <2.5 | <5.0 | 18.0 | 1.4 |
| Control | <2.5 | <2.5 | <5.0 | <0.3 | <0.3 |

for 24 h at 4°C, diluted goat anti-guinea pig gamma-globulin antibody was added and further incubation was continued for 24 h at 4°C. The bound antigen was separated from free [$^{125}$I]ET by centrifugation at 1,500 $g$ for 30 min. After the supernatant was decanted, the precipitant was counted with a gamma-counter.

*Gel permeation chromatography*

Extracts prepared from the spent media of two pancreatic cancer cell lines (KP1N and KP3) and HUVECs were chromatographed by using a Sephadex G-50 superfine column by the previously reported method (Kusuhara *et al.*, 1990). The column was calibrated with [$^{125}$I]ET-1, [$^{125}$I]BSA and [$^{125}$I]sodium iodide.

*Measurement of thrombomodulin in cell lysate and spent media*

Confluent monolayer or suspension cells grown in a 100 mm culture dish were solubilised with a total of 2 ml of 0.5% Triton X-100 in 2 mM phenylmethylsulphonyl fluoride, 0.15 M sodium chloride, 50 mM Tris-HCl, pH 7.5, for 1 h at 4°C. They were collected into 2 ml microfuge tubes, centrifuged and the TM content of the supernatant was measured by enzyme immunoassay. The above-mentioned buffer was used as a negative control. Spent media were prepared in the same manner as described elsewhere in this paper. Fresh medium supplemented with 5% FCS was used as a negative control. Enzyme immunoassay for TM was performed by the previously described method (Ishii *et al.*, 1990) with minor modifications. The detection limit was 0.3 ng ml$^{-1}$. The TM content of the cell lysate was first evaluated as ng per ml of lysate, then corrected according to the number of cells in the culture dish and reported as ng ml$^{-1}$ 10$^{-6}$ cells.

*Northern blot analysis*

Four pancreatic cancer cell lines were examined for expression of TM mRNA. After total cellular RNA was extracted by the acid guanidinium-phenol-chloroform method (Chomczynski & Sacchi, 1987), poly-A(+) RNA was selected with Oligotex dT-30 (Kuribayashi *et al.*, 1988). Gel electrophoresis and Northern blot hybridisation were performed as reported previously (Honda *et al.*, 1988) with minor modification. Briefly, 5 µg of mRNA per lane was separated on a 1% formaldehyde-agarose gel, and transferred to Hybond-N (Amersham). Hybridisation was performed at 42°C for 20 h in hybridisation buffer containing 50% formamide and 0.8 M sodium chloride. Washing was performed three times with 2 × SSC containing 0.1% SDS at 50°C for 10 min. For detecting TM mRNA, an oligodeoxyribonucleotide was synthesised and used as the probe. Its sequence was 5′-CAC CGA GGA GCG CAC TGT CAT TAG GTG GCC CCG CAG TCC GTC GCA GAT CTG ACT GGC ATT-3′, which is the anti-sense sequence for nucleotides 139-198 of TM (Suzuki *et al.*, 1987). It was labelled with [γ-$^{32}$P]ATP and used at a concentration of about 5 × 10$^6$ c.p.m. ml$^{-1}$. β-Actin mRNA running at about 2.0 kb was also examined to determine the integrity of these mRNA samples by the previously described method (Honda *et al.*, 1988).

*Immunostaining procedure for thrombomodulin*

After cells grown in chamber slides were fixed with 10% buffered formalin for 1 h, the slides were rinsed with phosphate-buffered saline (PBS) containing 3% BSA for 30 min. TM was detected by the avidin-biotin-peroxidase method by using a monoclonal antibody, TMMAb20 (Ishii *et al.*, 1990), as a primary antibody. The slides were incubated with TMMAb20 at a concentration of 1 µg ml$^{-1}$ in PBS containing 3% BSA for 6 h, and washed in PBS. Then a biotinylated anti-mouse IgG antibody was added as a second antibody at the recommended concentration and the slides were incubated with a solution of the avidin-biotinylated horseradish peroxidase complex for 30 min. Peroxidase was detected with diaminobenzidine used as a chromogen. Endogenous peroxidase was blocked with hydrogen peroxide in methanol. All procedures were performed at room temperature.

### Results

*Detection of tumour markers in the spent media*

The concentrations of the three tumour markers in the spent media of the 12 pancreatic cancer cell lines are shown in Table I. CA19-9 was detected in eight cell lines (67%). CEA and DUPAN-2 were detectable in four (33%) and three (25%) cell lines respectively.

*Endothelins in spent media*

As shown in Table II, immunoreactive (IR) ET-1 was detected in the spent media of 11 of the 12 pancreatic cancer cell lines (92%). The concentrations of IR-ET-1 ranged from 5.2 to 110 pM. HUVECs also produced a large amount of IR-ET-1, at a concentration of 410 pM. On the other hand, IR-ET-2 and IR-ET-3 were not detectable in any cell lines in this study, except that IR-ET-2 was detected in one cell line at a concentration of 8.0 pM. None of these three IR-ETs were detectable in fresh medium supplemented with 5% FCS. For malignant cells other than pancreatic cancer, there was no IR-ET at detectable level in any of the four cell lines examined (data not shown).

*Gel filtration studies*

The gel filtration patterns of the extracts prepared from the spent media of two pancreatic cancer cell lines and HUVECs are shown in Figure 1. The major peak was eluted at the position identical to that of synthetic ET-1.

*Thrombomodulin in the cell lysates and in the spent media*

The concentrations of TM in the cell lysate and in the spent media are shown in Table II. Cell lysates prepared from 11 pancreatic cancer cell lines (92%) contained various amounts of TM ranging from 0.31 to 150 ng ml$^{-1}$ 10$^{-6}$ cells. Furthermore, TM was also detected in the spent media of seven of those 11 cell lines (58%), especially in the cell lines containing relatively large amounts of TM in cell lysates. Only one cell line, MIAPaCa-2, which did not produce ET-1 either, did not produce a detectable amount of TM in its cell lysate. On the other hand, HUVECs expressed TM at a concentration of 18 ng ml$^{-1}$ 10$^{-6}$ cells. TM was not detected in buffer containing detergent and in fresh medium supplemented with 5% FCS. Of the other malignant cell types, A-549 cells contained TM at a concentration of 0.45 ng ml$^{-1}$ 10$^{-6}$ cells, but TM was not detected in cell lysates of the other three cell lines (data not shown).

*Expression of mRNA for thrombomodulin*

The autoradiogram of Northern blot analysis for TM mRNA in four pancreatic cancer cell lines is shown in Figure 2. A band with a molecular size of approximately 4.3 kb was detected in the ASPC-1 and BxPC-3 cell lines, which produced large amounts of TM. In contrast, there was no band in FA-6 and MIAPaCa-2, which produced only a small or undetectable amount of TM. β-Actin mRNA was expressed in all the cell lines tested.

*Immunostaining for thrombomodulin*

Positive staining for TM was obtained in all 11 preparations of pancreatic cancer cells (92%) whose lysates contained amounts of TM detectable by enzyme immunoassay. Positive staining was limited to the cell surface. In contrast, specific staining was not detected in MIAPaCa-2 cells, in which TM

was not detectable in the cell lysate by enzyme immunoassay. Typical examples of positive and negative staining cells are shown in Figure 3.

**Figure 1** Gel filtration patterns of IR-ET-1 extracted from conditioned media of pancreatic cancer cell lines and human umbilical vein endothelial cells (HUVEC). **a**, HUVEC; **b**, KP1N; **c**, KP3. Markers are shown at the top.

**Figure 2** Expression of TM mRNA in human pancreatic cancer cell lines. **a**, ASPC-1; **b**, BxPC-3; **c**, FA-6; **d**, MIAPaCa-2. 28S and 18S ribosomal RNA were used as molecular size markers.

**Figure 3** Cells stained by monoclonal antibody for thrombomodulin. **a**, ASPC-1; **b**, PSN-1; **c**, MIAPaCa-2 (original magnification × 840).

### Discussion

Twelve human pancreatic cancer cell lines were examined in the present study. We analysed the production and the release of three tumour markers in comparison to ET-1 and TM. CA19-9 and DUPAN-2 are relatively specific for pancreatic cancer, while CEA is not specific but is frequently produced by pancreatic cancer cells (Satake, 1991). In this study, eight of the 12 cell lines were found to produce CA19-9, and several produced CEA or DUPAN-2 as well. The four remaining cell lines were characterised mainly by morphological analysis (Lieber *et al.*, 1975; Yunis *et al.*, 1977; Owens *et al.*, 1979; Yamada *et al.*, 1986; Ikeda *et al.*, 1990), which suggested that they originated from pancreatic cancer.

Using these pancreatic cancer cell lines, we confirmed our previous observation that ET-1 is frequently produced in human pancreatic cancer cell lines (Kusuhara *et al.*, 1990). Further research revealed that the human genome has two other DNA sequences similar to ET-1 (Inoue *et al.*, 1989), and these two putative peptides were termed ET-2 and ET-3. Both are produced by several types of cells (Saida *et al.*, 1989; MacCumber *et al.*, 1990), but not by endothelial cells. With regard to ET-2 and ET-3, pancreatic cancer cells did not produce either, except for a minute amount of ET-2 in only one cell line. Based on these observations, it is reasonable to postulate that the pattern of expression of ET isoforms in human pancreatic cancer cell lines is similar to that of endothelial cells, which suggests that most of the pancreatic cancer cell lines share one of the properties of vascular endothelial cells (Kusuhara *et al.*, 1990): the production of ET-1.

We extended our study to determine whether another product of endothelial cells is produced by pancreatic cancer cell lines. TM is a membrane-bound glycoprotein with the ability to modulate blood coagulation (Esmon *et al.*, 1982; Maruyama *et al.*, 1985), and it is dominantly expressed in endothelial cells and the placenta. The following results clearly demonstrate that human pancreatic cancer cells frequently produce TM. First, TM was detected by specific enzyme immunoassay in almost all pancreatic cancer cell lysates and in the majority of the spent media. It is worth noting that pancreatic cancer cells produce a considerable amount of TM compared with that produced by human umbilical vein endothelial cells. Second, TM was also detected immunocytochemically in these cells. All of the cell lines in which TM was detected by enzyme immunoassay showed positive staining for TM. The distribution pattern of TM was compatible with that in endothelial cells (Esmon *et al.*, 1982; Maruyama *et al.*, 1985). Third, TM mRNA was expressed in two pancreatic cancer cell lines producing a large amount of TM. There are several studies on detection of TM in human primary tumours of vascular endothelial and syncytiotrophoblastic cell origin (Yonezawa *et al.*, 1987, 1988), malignant pleural mesothelioma (Collins *et al.*, 1992) and lung cancer (Tamura *et al.*, 1993). The fact that TM was detected in almost all pancreatic cancer cell lines suggests that TM could serve as a useful marker for primary human pancreatic carcinoma. Recent research also revealed increased plasma TM levels in pancreatic cancer patients (Lindahl *et al.*, 1993). Together with the possibility that it reflects coagulopathy often seen in pancreatic cancer patients, this phenomenon might be explained in part by the production of TM in pancreatic cancer tissue. This subject merits further investigation in the future.

The present study revealed that pancreatic cancer cells possess the biological characteristic of producing two products of endothelial cells, ET-1 and TM. Both ET-1 and TM were detected more frequently than tumour markers such as CA19-9, CEA and DUPAN-2. There seems to be no apparent correlation between ET-1 and/or TM production and the degree of differentiation of the tumours from which the cell lines were derived. Interestingly, placental syncytiotrophoblasts, which abundantly express TM as was the case for endothelial cells, do not produce ET-1 (Van Papendorp *et al.*, 1991). These facts raise the possibility that pancreatic cancer cells share some properties of vascular endothelial cells, rather than syncytiotrophoblasts. It might be that the pathological nature of pancreatic cancer cell growth, such as potent invasiveness (Furuta *et al.*, 1992), has some relationship to the mode of endothelial cell growth, which also seems to invade adjacent tissues (Furcht, 1986). In this context, the characteristics of pancreatic cancer cells presented here might be useful in investigating the biology of pancreatic cancer in the future.

The authors are particularly grateful to Mr J. Tsubouchi (Mitsubishi Gas Chemical Co, Ltd) for measurement of TM by enzyme immunoassay. We also thank Ms K. Otsubo and Ms M. Ebinuma for their excellent technical assistance. This investigation was supported in part by a Grant-in-Aid from the Ministry of Health and Welfare for Comprehensive 10-year Strategy of Cancer Control, and by the Special Coordination Funds from the Science and Technology Agency for Promoting Science and Technology, Japan. T. Oikawa is a Research Resident Fellow of the Foundation for Promotion of Cancer Research, Japan.

### References

CHOMCZYNSKI, P. & SACCHI, N. (1987). Single-step method of RNA isolation by acid guanidinium thiocyanate-phenol-chloroform extraction. *Anal. Biochem.*, **162,** 156-159.

COLLINS, C.L., ORDONEZ, N.G., SCHAEFER, R., COOK, C.D., XIE, S., GRANGER, J., HSU, P., FINK, L. & HSU, S. (1992). Thrombomodulin expression in malignant pleural mesothelioma and pulmonary adenocarcinoma. *Am. J. Pathol.*, **141,** 827-833.

ESMON, N.L., OWEN, W.G. & ESMON, C.T. (1982). Isolation of membrane-bound cofactor for thrombin-catalysed activation of protein C. *J. Biol. Chem.*, **257,** 859-864.

FURCHT, L.T. (1986). Critical factors controlling angiogenesis: cell products, cell matrix, and growth factors. *Lab. Invest.*, **55,** 505-509.

FURUTA, K., WATANABE, H. & IKEDA, S. (1992). Differences between solid and duct-ectatic types of pancreatic ductal carcinomas. *Cancer*, **69,** 1327-1333.

HONDA, S., YAMAGUCHI, K., SUZUKI, M., SATO, S., ADACHI, I., KIMURA, S. & ABE, K. (1988). Expression of parathyroid hormone-related protein mRNA in tumors obtained from patients with humoral hypercalcemia of malignancy. *Jpn J. Cancer Res.*, **79,** 677-681.

IKEDA, Y., EZAKI, M., HAYASHI, I., YASUDA, D., NAKAYAMA, K. & KONO, A. (1990). Establishment and characterization of human pancreatic cancer cell lines in tissue culture and in nude mice. *Jpn J. Cancer Res.*, **81,** 987-993.

IMANISHI, K., YAMAGUCHI, K., SUZUKI, M., HONDA, S., YANAIHARA, N. & ABE, K. (1989). Production of transforming growth factor-α in human tumor cell lines. *Br. J. Cancer*, **59,** 761-765.

INOUE, A., YANAGISAWA, M., KIMURA, S., KASUYA, Y., MIYAUCHI, T., GOTO, K. & MASAKI, T. (1989). The human endothelin family: three structurally and pharmacologically distinct isopeptides predicted by three separate genes. *Proc. Natl Acad. Sci. USA*, **86,** 2863-2867.

ISHII, H., NAKANO, M., TSUBOUCHI, J., ISHIKAWA, T., UCHIYAMA, H., HIRAISHI, S., TAHARA, C., MIYAJIMA, Y. & KAZAMA, M. (1990). Establishment of enzymeimmunoassay of human thrombomodulin in plasma and urine using monoclonal antibodies. *Thromb. Haemostasis*, **63,** 157-162.

KURIBAYASHI, K., HIKATA, M., HIRAOKA, O., MIYAMOTO, C. & FURUICHI, Y. (1988). A rapid and efficient purification of poly(A)-mRNA by oligo(dT) 30-latex. *Nucleic Acids Res. Symposium Series*, **19,** 61-64.

KUSUHARA, M., YAMAGUCHI, K., NAGASAKI, K., HAYASHI, C., SUZAKI, A., HORI, S., HANDA, S., NAKAMURA, Y. & ABE, K. (1990). Production of endothelin in human cancer cell lines. *Cancer Res.*, **50,** 3257-3261.

LIEBER, M., MAZETTA, J., NELSON-RESS, W., KAPLAN, M. & TODARO, G. (1975). Establishment of a continuous tumor-cell line (PANC-1) from a human carcinoma of the exocrine pancreas. *Int. J. Cancer*, **15,** 741-747.

LINDAHL, A.K., BOFFA, M.C. & ABILDGAARD, U. (1993). Increased plasma thrombomodulin in cancer patients. *Thromb. Haemostasis*, **69,** 112-114.

MACCUMBER, M.W., ROSS, C.A. & SNYDER, S.H. (1990). Endothelin in brain: receptors, mitogenesis, and biosynthesis in glial cells. *Proc. Natl Acad. Sci. USA*, **87,** 2359-2363.

MARUYAMA, I., BELL, C.E. & MAJERUS, P.W. (1985). Thrombomodulin is found on endothelium of arteries, veins, capillaries, lymphatics, and on syncytiotrophoblast of human placenta. *J. Cell. Biol.*, **101,** 363-371.

MORI, M., YAMAGUCHI, K., HONDA, S., NAGASAKI, K., UEDA, M., ABE, O. & ABE, K. (1991). Cancer cachexia syndrome developed in nude mice bearing melanoma cells producing leukemia-inhibitory factor. *Cancer Res.*, **51,** 6656-6659.

MOTOYAMA, T., HOJO, H. & WATANABE, H. (1986). Comparison of seven cell lines derived from human gastric carcinomas. *Acta Pathol. Jpn*, **36,** 65-83.

NAGATA, N., AKATSU, T., KUGAI, N., YASUTOMO, Y., KINOSHITA, T., KOSANO, H., SHIMAUCHI, T., TAKATANI, O. & UEYAMA, Y. (1989). The tumor cells (FA-6) established from a pancreatic cancer associated with humoral hypercalcemia of malignancy: a simultaneous production of parathyroid hormone-like activity and transforming growth factor activity. *Endocrinol. Jpn*, **36,** 75-85.

ODELL, W.D. & APPLETON, W.S. (1992). Humoral manifestations of cancer. In *Williams Textbook of Endocrinology*, Wilson, J.D. & Foster, D.W. (eds) pp. 1599–1617. W.B. Saunders: Philadelphia.

OWENS, R.B., SMITH, H.S., NELSON-RESS, W.A. & SPRINGER, E.L. (1979). Epithelial cell cultures from normal and cancerous human tissues. *J. Natl Cancer Inst.*, **56**, 843–849.

SAIDA, S., MUTSUI, Y. & ISHIDA, N. (1989). A novel peptide, vasoactive intestinal contractor, of a new (endothelin) peptide family. *J. Biol. Chem.*, **264**, 14613–14616.

SATAKE, K. (1991). Diagnosis of pancreatic cancer. *Int. J. Pancreatol.*, **9**, 93–98.

SCHREY, M.P., PATEL, K.V. & TEZAPSIDIS, N. (1992). Bombesin and glucocorticoids stimulate human breast cancer cells to produce endothelin, a paracrine mitogen for breast stromal cells. *Cancer Res.*, **52**, 1786–1790.

SHIMOYAMA, M. (1975). SEKI strain (in Japanese). In *In vitro Culture of Human Cancer Cells*, Oboshi, S. & Sugano, H. (eds) pp. 208–215. Asakura Shoten: Tokyo.

SUZUKI, K., KUSUMOTO, H., DEYASHIKI, Y., NISHIOKA, J., MARUYAMA, I., ZUSHI, M., KAWAHARA, S., HONDA, G., YAMAMOTO, S. & HORIGUCHI, S. (1987). Structure and expression of human thrombomodulin, a thrombin receptor on endothelium acting as a cofactor for protein C activation. *EMBO J.*, **6**, 1891–1897.

TAMURA, A., MATSUBARA, O., HIROKAWA, K. & AOKI, N. (1993). Detection of thrombomodulin in human lung cancer cells. *Am. J. Pathol.*, **142**, 79–85.

VAN PAPENDORP, C.L., CAMERON, I.T., DAVENPORT, A.P., KING, A., BARKER, P.J., HUSKISSON, N.S., GILMOUR, R.S., BROWN, M.J. & SMITH, S.K. (1991). Localization and endogenous concentration of endothelin-like immunoreactivity in human placenta. *J. Endocrinol.*, **131**, 507–511.

YAMADA, H., YOSHIDA, T., SAKAMOTO, H., TERADA, M. & SUGIMURA, T. (1986). Establishment of a human pancreatic adenocarcinoma cell line (PSN-1) with amplifications of both c-myc and activated c-Ki-ras by a point mutation. *Biochem. Biophys. Res. Commun.*, **140**, 167–173.

YAMASHITA, J., OGAWA, M., EGAMI, H., MATSUO, S., KIYOHARA, H., INADA, K., YAMASHITA, S. & FUJITA, S. (1992). Abundant expression of immunoreactive endothelin-1 in mammary phyllodes tumor: possible paracrine role of endothelin-1 in the growth of stromal cells in phyllodes tumor. *Cancer Res.*, **52**, 4046–4049.

YANAGISAWA, M., KURIHARA, H., KIMURA, S. TOMOBE, Y., KOBAYASHI, M., MITSUI, Y., YAZAKI, Y., GOTO, K. & MASAKI, T. (1988). A novel potent vasoconstrictor peptide produced by vascular endothelial cells. *Nature*, **332**, 411–415.

YONEZAWA, S., MARUYAMA, S., SAKAE, K., IGATA, A., MAJERUS, P.W. & SATO, E. (1987). Thrombomodulin as a marker for vascular tumors. *Am. J. Clin. Pathol.*, **88**, 405–411.

YONEZAWA, S., MARUYAMA, I., TANAKA, S., NAKAMURA, T. & SATO, E. (1988). Immunohistochemical localization of thrombomodulin in chorionic diseases of the uterus and choriocarcinoma of the stomach. *Cancer*, **62**, 569–576.

YUNIS, A.A., ARIMURA, G.K. & RUSSIN, D.J. (1977). Human pancreatic carcinoma (MIAPaCa-2) in continuous culture: sensitivity to asparaginase. *Int. J. Cancer*, **19**, 128–135.